# Enhancer identification in mouse embryonic stem cells using integrative modeling of chromatin and genomic features

**DOI:** 10.1186/1471-2164-13-152

**Published:** 2012-04-26

**Authors:** Chih-yu Chen, Quaid Morris, Jennifer A Mitchell

**Affiliations:** 1 Department of Cell and Systems Biology, University of Toronto, 25 Harbord Street, Toronto, ON, , M5S 3G5, Canada; 2 Donnelly Centre for Cellular and Biomolecular Research, University of Toronto, Toronto, Canada

**Keywords:** Enhancer, Embryonic stem cells, Transcription factor, ChIP-Seq, Histone methylation, Regulation of gene expression

## Abstract

**Background:**

Epigenetic modifications, transcription factor (TF) availability and differences in chromatin folding influence how the genome is interpreted by the transcriptional machinery responsible for gene expression. Enhancers buried in non-coding regions are found to be associated with significant differences in histone marks between different cell types. In contrast, gene promoters show more uniform modifications across cell types. Here we used histone modification and chromatin-associated protein ChIP-Seq data sets in mouse embryonic stem (ES) cells as well as genomic features to identify functional enhancer regions. Using co-bound sites of OCT4, SOX2 and NANOG (co-OSN, validated enhancers) and co-bound sites of MYC and MYCN (limited enhancer activity) as enhancer positive and negative training sets, we performed multinomial logistic regression with LASSO regularization to identify key features.

**Results:**

Cross validations reveal that a combination of p300, H3K4me1, MED12 and NIPBL features to be top signatures of co-OSN regions. Using a model from 10 signatures, 83% of top 1277 putative 1 kb enhancer regions (probability greater than or equal to 0.8) overlapped with at least one TF peak from 7 mouse ES cell ChIP-Seq data sets. These putative enhancers are associated with increased gene expression of neighbouring genes and significantly enriched in multiple TF bound loci in agreement with combinatorial models of TF binding. Furthermore, we identified several motifs of known TFs significantly enriched in putative enhancer regions compared to random promoter regions and background. Comparison with an active H3K27ac mark in various cell types confirmed cell type-specificity of these enhancers.

**Conclusions:**

The top enhancer signatures we identified (p300, H3K4me1, MED12 and NIPBL) will allow for the identification of cell type-specific enhancer regions in diverse cell types.

## Background

Chromatin immunoprecipitation followed by massively parallel sequencing (ChIP-Seq) has enabled genome-wide investigation of chromatin features and epigenetic modifications within the non-coding regions of mammalian genomes in high resolution [1]. ChIP-Seq provides the opportunity to characterise and begin to understand on a genome-wide scale how genes are regulated in a cell-type specific manner by sequence-specific DNA-binding transcription factors (TFs). However, identifying regulatory regions within the genome and linking these regions to the regulation of specific genes remains a challenge.

Distal regulatory elements have been identified which regulate gene transcription from several kilobases (kb) away and have even been found to regulate genes located on separate chromosomes [2-4]. Functional characterisation of these regulatory elements can be done by identifying bound TFs and investigating whether or not they act as enhancers, increasing transcription of a gene in a position and orientation independent manner. ChIP-Seq analysis for several TFs has revealed a significant fraction (40–60%) of the binding sites for most TFs are located in intergenic regions >10 kb from transcription start sites (TSSs) of annotated genes [5-7]. In addition, enhancer regions are associated with significant epigenetic differences between cell types, while gene promoters show more uniform modifications across different cell types [8,9]. These findings suggest that enhancers, which can be located at great distances from the genes they regulate, play a larger role in regulating tissue-specific gene expression than the sequences proximal to gene promoters. Moreover, mutations in DNA sequences of distant-acting enhancers contribute to various diseases [10], further stressing their importance in regulating gene expression.

Prior to the availability of ChIP-Seq and ChIP-chip data, computational approaches based solely on genomic sequences were used to identify enhancer regions. Initially these approaches compared the genomic sequence with TF binding motifs represented by position specific scoring matrices (PSSM) from TRANSFAC [11] and JASPAR [12]. TF motif clustering and comparative genomics improved the predictive power of these approaches [13-16]. In addition, intergenic regions with high sequence conservation between human and *Fugu* or ultra-conserved regions between human-mouse-rat (>200 bp of 100% identity) are predictive of regulatory regions involved in conserved processes such as embryonic development [17,18]. As many enhancer regions regulate the expression of genes in a tissue-specific manner and can be located at great distances from the genes they regulate, the link between cell-type and active enhancers is lacking in purely sequenced based approaches. Using ChIP-Seq approaches several different methods of identifying enhancers have been applied including: enrichment of mono-methylated lysine 4 of histone H3 (H3K4me1) and depletion of tri-methylated lysine 4 of histone H3 (H3K4me3) [19], binding of the co-activator p300 (also known as EP300) [9], intergenic RNAPII (RNA polymerase II) phosphorylated at serine 5 on the C-terminal domain (RNAPII-ser5) [20], multiple transcription factor bound loci (MTL) [5], and a combination of these features [21] have been used to identify enhancers within a target cell type. Additional chromatin associated proteins have been identified at enhancers including members of the mediator (MED1, MED12) and cohesin (SMC1A, SMC3, NIPBL) complexes [22].

These different approaches show variable success at predicting enhancers with *in vivo* activity. For example, 47% (246/528) of human genomic regions predicted by sequence conservation were confirmed as enhancers in a transgenic mouse assay [17,18]. The prediction was significantly enhanced to 87% (75/86) when using only p300 high throughput chromatin immunoprecipitation sequencing (ChIP-Seq) binding sites from mouse forebrain, midbrain and limb cells [9]. Heintzman et al. developed a motif-independent model for identifying and distinguishing promoters and enhancers using histone modification profiles [19]. They observed H3K4me1 enrichment and H3K4me3 depletion at p300 binding sites and then used this signature to identify putative enhancers in 5 human cell lines. Their enhancer predictions were supported by DNase I hypersensitivity, binding of p300, or binding of the mediator protein MED1 63.5% of the time. Taking the MTL approach, using overlapping regions of three major pluripotency TFs, OCT4, SOX2, NANOG, Chen et al. 2008 generated enhancer candidates in mouse embryonic stem (ES) cells and tested 25 of these regions for enhancer activity [5]. All 25 regions displayed ES cell specific enhancer activity suggesting the MTL approach is highly predictive of functional enhancer regions. However, enhancer activities depend on the specific TFs occupying the MTL, as all 8 co-MYC associated MTL were found to have little or no enhancer activity [5]. In addition, the MTL approach requires prior knowledge of relevant regulatory TFs and the generation of unique ChIP-Seq data sets for each cell type.

Although previous approaches showed promising performance, a potential issue with non-integrated approaches is that each marker may be an incomplete representation of the relevant enhancers in a particular cell type. For example, although sequence conservation is frequently used to identify regulatory elements, ultra-conservation has been reported to identify only a small subset of developmentally related enhancers, specifically, those involved in development of the nervous system [18]. Furthermore, there is variation in the degree of conservation at enhancers; a large population of validated heart enhancers are less deeply conserved in vertebrate evolution [23]. Although found at enhancer regions in different cell types, p300 is reported to mark only a subset of enhancers in heart [24]. Histone modifications perhaps represent a more widely applicable enhancer signature, though by inspection they appear to mark the genome in a broad manner. The incomplete representation of enhancers by each feature and the broad signatures associated with histone modifications emphasize the need to integrate these features within the same cell type and evaluate their importance for enhancer prediction.

In this study we used 31 public high throughput mouse ES cell RNA-Seq and ChIP-Seq datasets, as well as 5 genomic features to identify the characteristics most predictive of enhancers. We applied multinomial logistic regression with LASSO regularization [25,26] to identify key enhancer signatures, predict functional enhancers, and subsequently identify motif enrichment in predicted enhancers. Through an initial assessment of enhancer markers, we highlight the importance of feature selection. Furthermore, LASSO regularization ranked the predictability of signatures necessary for enhancer classification with p300 being the most predictive followed by H3K4me1 and MED12. Predicted enhancers showed significant association with MTL indicating functionality and identified previously validated enhancers. In addition, a supervised motif enrichment test on putative enhancer regions using Clover [27] confirmed our ability to identify known TFs centrally involved in ES cell transcriptional regulatory networks.

## Results

### Feature selection improves enhancer prediction

**Figure 1:**
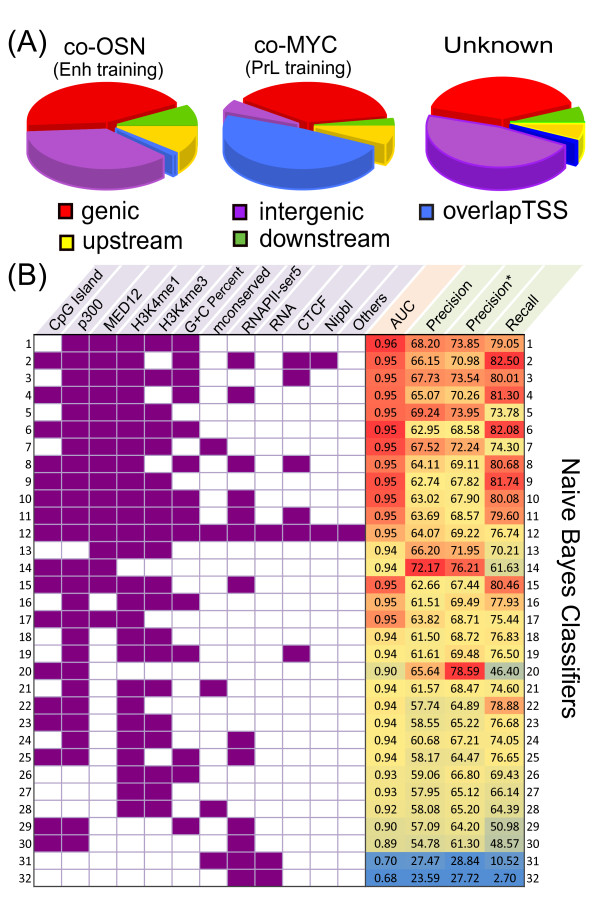
**Assessing feature combinations as enhancer signatures with cross validation using Naive Bayes classifications**. **(A)** Pie charts representing the genomic distributions of the co-OSN and co-MYC, unknown training sets. Intergenic regions are defined to be regions ≥ 10 kb away from the closest TSS or transcription end site; whereas upstream regions are regions within 10 kb upstream of TSSs. **(B)** The first 11 columns depict the features used in each given row (Naive Bayes classifier) and the 12th (Others) column represents the rest of the features listed in Table 1. The capability of each classifier in categorizing co-OSN regions (Enh training set) from co-MYC regions (PrL training set) and unknown is assessed using 10-fold cross validation. The last four columns listing the area under ROC curve (AUC), precision, modified precision (precision*) and recall values are color-coded with red indicating good model performance and blue indicating poor performance. Naive Bayes classifiers with different feature combination are sorted by the average ranking of the four indices.

To identify and validate enhancer regions active in mouse ES cells we used ChIP-Seq data sets from mouse ES cells including: 12 transcription factors (TFs) [5,28], 8 histone modifications [29], 3 polymerase occupancy [30], and 7 chromatin associated proteins [5,22] (Table 1). In addition to these ChIP-Seq data sets, genomic features including CpG islands, GC content, SNP, repeat regions, and PhastCons most conserved regions [31] were also incorporated in the model. To prevent cell type specificity in the features used for enhancer identification, only histone modifications and ubiquitously expressed non-TF features were evaluated as enhancer markers. The TF data sets were used for either training or validation purposes. As 25/25 regions co-bound by the pluripotency transcription factors OCT4, SOX2 and NANOG (co-OSN) in ChIP-Seq data were shown to have enhancer activity [5], co-OSN regions were used as the enhancer positive training set (Enh). In contrast, 8/8 regions co-bound by MYC and MYCN (co-MYC) had limited or no enhancer activity [5]. As co-MYC regions showed a strong tendency to be located close to annotated TSSs (Figure 1A) we termed this enhancer negative training set promoter-like (PrL, see Methods). We also included an “unknown” training set of 5000 regions randomly drawn from non co-OSN and non co-MYC regions of the genome.

**Table 1:** Datasets used in the integrative modeling

**Data**	**Data Type**	**Cell Line**	**Purpose**	**Accession**	**Ref**
RNA	RNA-Seq	V6.5 cells	Feature	GSE20851	[32]
Histone modifications (H3, H3K4me1, H3K4me2, H3K4me3, H3K36me3, H4K20me3, H3K27me3, H3K9me3) and RNAPII	ChIP-Seq	V6.5 cells	Feature	GSE11172 GSE12241	[29]
RNAPII-ser2, RNAPII-ser5	ChIP-Seq	V6.5 cells	Feature	GSE20530	[30]
SMC1A, SMC3, MED12, MED1, NIPBL	ChIP-Seq	V6.5 cells	Feature	GSE22562	[22]
p300	ChIP-Seq	E14 cells	Feature	GSE11431	[5]
CTCF	ChIP-Seq	V6.5 cells	Feature	GSE18699	[28]
CpG islands, GC content, SNP, repeat regions and PhastCons most conserved regions	BED files	--	Feature	mm9	[31,33-35]
OCT4, SOX2, NANOG, MYC, MYCN	ChIP-Seq	E14 cells	Training sets	GSE11431	[5]
KLF4, STAT3, SMAD1, E2F1, TFCP2L1, ZFX, ESRRB	ChIP-Seq	E14 cells	MTL analysis	GSE11431	[5]

The RNA-Seq, ChIP-Seq and genomic feature data sets used in the study are listed and cited in the table. Columns represent the cell line of mouse ES cells used (Cell Line), the usage of the data (Purpose), the NCBI GEO accession number (Accession), and the reference in which the data was generated (Ref). MTL: multiple transcription factor bound loci.

In our model we classified 1 kb genomic bins into 3 categories: enhancer positive (Enh), promoter-like (PrL) and unknown. As multiple combinations of features have been used to predict enhancers, we performed Naive Bayes classifications using various combinations of features. Assessment of the model was carried out using four indices, area under curve (AUC), precision, modified precision, and recall (detailed in Methods), using 10 fold cross validation on the training set data (Figure 1B). The classifiers were ranked by the average ranking of the four indices. We used this analysis to make a number of observations about the predictive value of various markers of enhancers within the context of other possible features. First, we noted that the Naive Bayes classifier using all features (rank 12) does not generate the best classification. Such classification bias can be caused by feature redundancy and introduction of noise by non-informative features. Second, although p300 is extensively used to identify enhancers and has the highest modified precision in combination with CpG islands, the recall is low which is likely caused by the incomplete representation of enhancers by p300 (rank 20). Classifiers using previous markers, such as H3K4me1 and H3K4me3 as well as RNAPII-ser5 and RNA, also performed poorly (rank 27 and 32). Third, adding GC content, an informative genomic feature, improves precision and recall (comparing rank 5 to 1). Fourth, the recall of enhancers can be over 80 percent with informative features (rank 2 and 4). Finally, we observed that although sequence conservation has been previously used to predict enhancers, adding most conserved regions into feature combinations can worsen the model prediction (rank 5 to 7; 18 to 21), which is likely due to non-exclusive representation of enhancers in conserved regions.

### Ranking enhancer signatures

**Figure 2:**
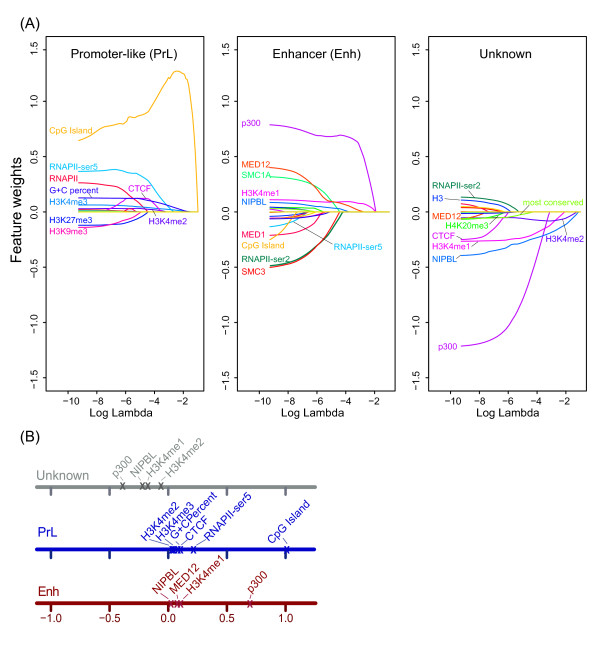
**Feature ranking determined using multinomial logistic regression with LASSO regularization. (A)** Feature weights in each class with respect to log lambda, which is a penalty factor to shrink feature weights. Weights of features less discriminative of the three categories shrink to 0 as lambda is increased. Top ranking features are those with non-zero weights at high lambda values: p300, H3K4me1 and MED12 for Enh group; CpG islands, H3K4me3, G + C percent and RNAPII-ser5 for PrL groups. **(B)** Signatures used in the model chosen from cross validation (log lambda = −4) are shown in each category with their degree of contribution.

The Naive Bayes classification approach revealed the importance of feature selection and allowed us to determine the most appropriate combination of features for optimal enhancer identification, however, each feature is assumed to contribute independently. As some features might be partially or fully redundant (e.g. histone modifications) and some may add no or little predictive value, we modified our approach and used a LASSO regularized multinomial logistic regression model [25,26] to assess features systematically and obtain their rank contributions to the classification of enhancers. LASSO regularization [25,26] introduces a lambda penalty factor to shrink feature weights, so uninformative or redundant features can be assigned zero weights and not impact classification. Furthermore, regression models can detect and down-weight highly correlated (and likely redundant) features. Feature weights corresponding to specific log lambda values for each category are shown in Figure 2A. By increasing the penalty parameter lambda, weights of less informative features for each category shrink to zero; whereas weights of informative features remain non-zero. Through LASSO regularization we identified the most positively predictive features for Enh regions, p300 and H3K4me1, which have been used for enhancer prediction in other studies [9,19,36]. The component of the mediator complex, MED12, and the cohesion loading factor, NIPBL, are ranked third and fourth. Both have been shown to associate with enhancers involved in chromatin looping to promoter regions [22]. Features that best categorize PrL regions are CpG islands, H3K4me3, G + C percent and RNAPII-ser5 all in agreement with promoter characteristics. Ten features were selected to classify the 1 kb bins into three categories in our model (log lambda = −4, Figure 2B).

### Classified enhancer and promoter-like candidates

A total of 19200 1 kb regions were predicted to be Enh, 67672 were predicted to be PrL, and 2567872 regions were predicted as unknown using the LASSO regularized model ( Additional file 1: Table S1). Of the 1291 co-OSN training regions 922 were classified as Enh in the model, while 4054 of 4465 co-MYC training regions were classified as PrL. All luciferase-validated enhancer positive regions from Chen et al. 2008 [5] are predicted positive whereas, all luciferase-validated enhancer negative regions are predicted PrL in our model. As Enh candidates are ranked by probability, more stringent thresholds can be applied to gain higher confidence enhancer candidates. Applying a stringent threshold of greater than 0.8 probability, 24 of 25 previously validated enhancer regions were predicted Enh and 7 of 8 previously identified negative regions were predicted PrL. In addition, the log probabilities of putative enhancer candidates are significantly correlated (ρ = 0.44, *p* = 0.0002) to an independent luciferase assay data set in which 67 regions bound by CHD7 were tested for enhancer activity [37]. A heatmap of the top 50 Enh and PrL candidates demonstrates that the Enh and PrL signature features are separated with hierarchical clustering except for H3K4me2, the lowest ranked feature in the PrL category ( Additional file 2: Figure S1). The probability and modeling approach, in contrast to an overlapping peak approach, allows variations in features within the predicted category.

**Figure 3:**
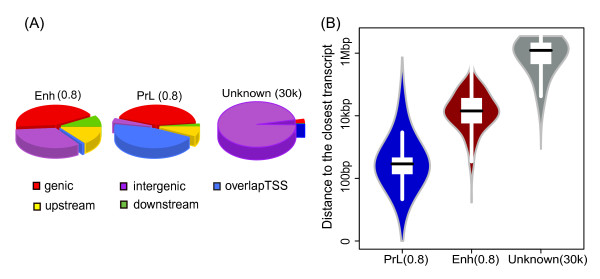
**Genomic distribution of enhancer and promoter-like candidates. (A)** Pie charts representing the genomic distributions of the high confidence Enh and PrL candidates (prob ≥ 0.8) and top 30 k unknown candidates. Intergenic regions are defined to be regions ≥ 10 kb away from the closest TSS or transcription end site; whereas upstream regions are regions within 10 kb upstream of TSSs. **(B)** Violin plots demonstrating the distances to TSS of the closest transcript for each high probability set (Enh and PrL: prob ≥0.8; unknown: top 30,000).

We examined the distribution of Enh and PrL candidates in the genome and found that high probability (≥0.8) Enh and PrL candidates are distributed relative to annotated genes similarly to the training data (Figure 3A). As expected more of the top Enh candidates are located in intergenic regions (33%, *p* < 2.2 × 10^−16^) than top PrL candidates, which more frequently overlap TSSs. To examine the distribution of Enh and PrL candidates more specifically with respect to TSSs we calculated the distance to the closest TSS for each candidate. Enh candidates tend to be further away from TSSs compared to PrL candidates (*p* < 2.2 × 10^−16^, Figure 3B). Distributions of the entire set of the three categories are provided in Additional file 3: Figure S2.

### Enhancer and promoter-like candidates coordinately regulate gene expression

**Figure 4:**
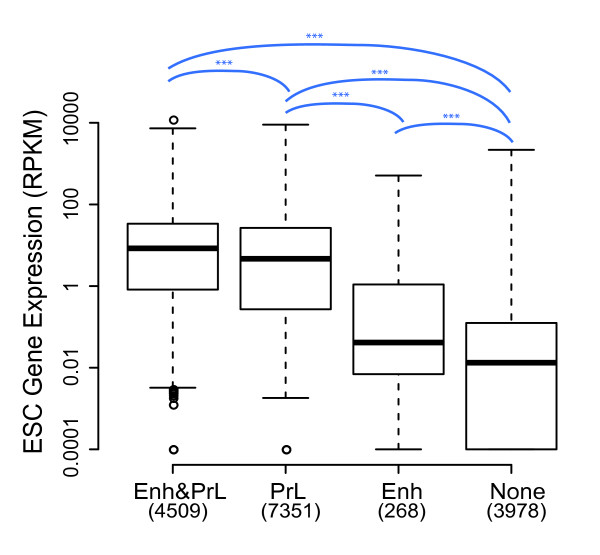
**Enhancer and promoter-like candidates co-ordinately regulate gene expression.** Boxplots of ES cell gene expression of the genes closest to various subsets of Enh and PrL candidates. Enh&PrL denotes genes with at least one of both Enh and PrL closest to their TSSs; PrL set denotes genes with PrL but not Enh closest to them and vice versa for Enh set; None set denotes genes without any Enh and PrL closest to them. The numbers in brackets below each set show the counts of genes within the category. The y-axis denotes gene expression represented by RPKM plotted on a log scale. Distributions of gene expression are all significantly higher compared to sets on their right (*** *p*-values <10^−7^).

To assess the regulatory potential of the Enh and PrL candidates in ES cells, we assigned candidates to the closest gene TSSs and compared the ES cell gene expression distribution among subsets of genes: associated with both Enh and PrL, either PrL or Enh only, and genes without an associated Enh or PrL candidate (denoted as Enh&PrL, PrL, Enh and None, respectively) (Figure 4). Distributions of gene expression in all categories containing an Enh or PrL candidate are significantly higher than those with no associated candidate. While we found that PrL alone conferred significantly higher expression than Enh alone (*p* = 4.1 × 10^−46^), Enh&PrL genes showed significantly higher expression than that of PrL-only genes (*p* = 2.3 × 10^−18^). These findings suggest that Enh and PrL candidates coordinately regulate transcription of a subset of target genes in mouse ES cells, while other genes are regulated solely by PrL signatures. It is important to note that Enh candidates are expected to be more difficult to assign to the correct gene(s) as they are often located in intergenic regions and may in fact not regulate the closest gene in the linear genome. Regardless, we observe the presence of a candidate enhancer is associated with increased levels of expression in addition to the presence of a promoter candidate.

Short intergenic transcripts have been found associated with enhancers in neurons [38], while the human β-globin distal locus control region (LCR) is associated with long, cell cycle-regulated intergenic transcripts [39,40]. We investigated the overlap between lincRNAs (large intergenic non-coding RNA) identified in ES cells and our Enh and PrL categories. We found that only 135 out of 2127 lincRNAs (0.7% total Enh) from Guttman et al. 2009 [41] overlapped predicted Enh regions and only 22 (1.7% high probability Enh) overlapped high probability Enh candidates (prob ≥ 0.8). A larger proportion overlapped PrL regions, 501 (3.1% total PrL) and 233 (2.3% high probability PrL) of the 2127 lincRNAs overlapped the total and top (prob ≥ 0.8) PrL candidates, respectively. In addition, the proximity of Enh candidates to protein coding transcripts is significantly greater than the proximity to these lincRNAs (*p* < 2.2 × 10^−16^). The enhancer associated transcripts identified by Kim et al. 2010 [38] are generally short, <2 kb in length, and lincRNAs were defined as at least 5 kb in length. These may therefore represent functionally different types of intergenic non-coding transcripts.

In order to probe the functional significance of the genes regulated by Enh or PrL, we investigated functional enrichment in these categories using DAVID [42,43] followed by clustering of functions with significant numbers of shared genes using Enrichment map [44]. The Enh set is significantly enriched with DNA binding, transcription regulating activities and stem cell development (FDR = 1.2 × 10^−06^, 0.0018, and 0.03 respectively), and the genes include several TFs involved in ES cell transcriptional regulation: *Oct4, Myc, Mycn, Sox2, Esrrb, Phc1 and Zic3* ( Additional file 4: Figure S3). In contrast the PrL candidates are enriched with a wide variety of molecular functions in addition to DNA binding, such as RNA binding and processing, translation, and chromatin organization (all FDR < 1.0 × 10^−8^) indicating these genes are associated with more basal cellular functions. This indicates that, in addition to promoters, Enh candidates play a significant role in regulating the ES cell transcriptional program to maintain pluripotency.

### Enhancer candidates are bound by multiple transcription factors in ES cells

**Figure 5:**
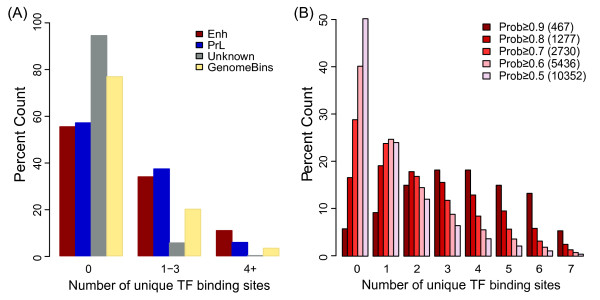
**Enhancer candidates overlap multiple transcription factor bound loci.(A)** Percentage overlap of Enh, PrL, Unknown and all 1 kb genome bins to TF bound regions in three categories (0, 1–3 or 4+ TFs). Generated by comparison to ChIP-Seq data for 7 ES cell-expressed TFs (excluding OCT4, SOX2, NANOG, MYC and MYCN). The Enh candidates are significantly enriched with MTL in comparison to the PrL and unknown sets as well as whole genome bins with *p*-values <0.001. **(B)** Percentage overlap of Enh candidates with TF bound regions using various enhancer probability thresholds. The numbers in brackets beside enhancer probability thresholds show the number of regions predicted to be Enh using the indicated threshold.

As we initially trained our classifier based on the binding of either co-OSN (Enh signature) or co-MYC (PrL signature), we wanted to determine whether we also identified regions bound by additional TFs. Single TF binding sites are found at many locations in the genome while MTL are more limited and have been shown to be associated with increased regulatory activity [5,24]. We examined the association of our Enh and PrL candidates with ES cell-expressed TFs using 7 ChIP-Seq data sets (Table 1, excluding the OCT4, SOX2, NANOG, MYC and MYCN training data). We found that the Enh candidates were most highly and significantly enriched with MTL (≥4 TFs) compared to both the PrL and unknown categories, and overall genomic bins (*p* < 10^−30^; Figure 5A). Of the 1277 top Enh candidate regions (prob ≥ 0.8), 1065 of these overlapped with at least one of the 7 TF binding sites. It is noteworthy that although Enh candidates tend to be further away from TSSs compared to PrL candidates, the Enh set is significantly more enriched in MTL than the PrL set. This is in agreement with the observation that a significant proportion of individual TF bound regions are located within the intergenic regions of the genome [5-7]. We also investigated the effect of increasing the enhancer probability threshold on the proportion overlapping MTL (Figure 5B). With increasing Enh probability we found an increase in the proportion of candidates associated with more bound TFs. This significant association of Enh candidates with MTL further supports the regulatory functionality of these regions.

### ES cell enhancer candidates

We next looked at how well the model predicts previously identified mouse ES cell enhancer regions associated with mediator and cohesin proteins and shown to form chromatin loops with the nearby gene TSSs [22]. These tissue-specific promoter-interacting enhancer regions upstream of *Oct4* (*Pou5f1)NanogPhc1* and *Lefty1* are all predicted Enh with probabilities greater than 0.8 ( Additional file 5: Figure S4). Moreover, several significant TF binding peaks overlap with these enhancers. Although the aligned TF peaks at the *Oct4* upstream enhancer are located at the boundary of two 1 kb genome bins, our model predicted both sides with high probability (prob = 0.9487 and 0.8363). In addition to the *Lefty1* promoter-interacting enhancer, we identified novel contiguous enhancer regions over 3–5 kb upstream of *Lefty2* (prob = 0.9107 and 0.8999).

**Figure 6:**
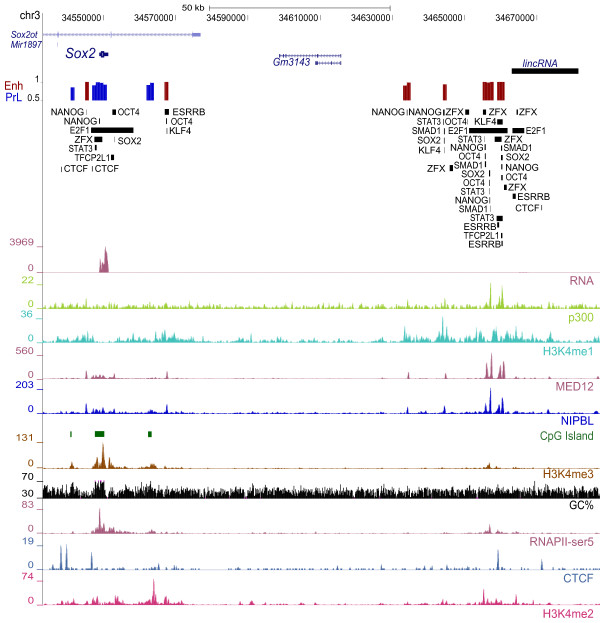
**Novel putative enhancer regions around*Sox2*.** Plot showing novel putative enhancers around the *Sox2* gene. The Enh and PrL probabilities of 1 kb bins are shown in red and blue bars, respectively. Only bins with probabilities greater than 0.8 are displayed for higher stringency, and the y-axis scale is from 0.5 to 1. A lincRNA approximately 100 kb downstream of Sox2 near the distal enhancer cluster is shown in black. Transcription factors peaks identified using the SISSRs algorithm are illustrated in rectangle boxes to demonstrate overlaps of the enhancers with TFs. Coverage plots of RNA-Seq data in ES cells and used features are shown at the bottom.

We also identified high probability Enh candidates surrounding the *Sox2* gene and around 100 kb downstream (Figure 6). In addition to the role SOX2 plays in regulating the transcriptional program in ES cells, *Sox2* is also a key neurodevelopmental gene, and multiple *Sox2* enhancers have been identified in various different cell types ranging from ES to neural precursor and lens epithelial cells [45-50]. The evolutionary conserved SRR1 enhancer, 4 kb upstream of *Sox2*, has been shown to enhance the expression of a reporter gene by 10 fold in ES cells and overlaps a high confidence Enh candidate (prob = 0.9956) [47,48,50], while the second validated enhancer 4 kb downstream of *Sox2*, SRR2, overlaps partially with a lower confidence Enh (prob = 0.6301; not shown). In addition to these previously validated enhancers, we identified a cluster of eight high confidence enhancers downstream of *Sox2* overlapping multiple TF peaks as well as p300 and MED12 peaks. Notably, the furthest downstream enhancer region is ranked in the top 4 with an Enh probability of 1. Located between the Sox2 gene and the enhancer cluster is a RefSeq transcript *Gm3143m* which is not expressed in ES cells and therefore unlikely to be regulated by the cluster of eight high confidence enhancers. We also noted an expressed lincRNA immediately downstream of the distal enhancer cluster ( Additional file 6: Figure S5 shows the expression of the lincRNA).

**Figure 7:**
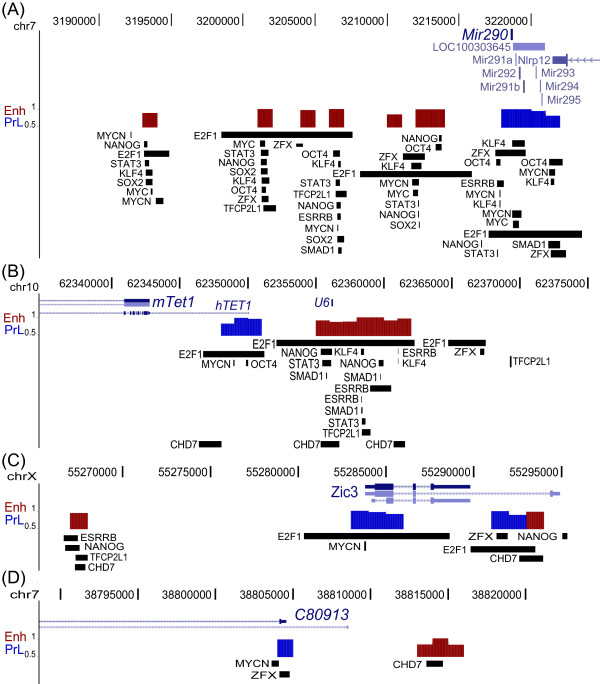
**Novel putative enhancer regions.** Plot showing novel putative enhancers around *miR-290***(A)***, Tet1*/*U6***(B)***, Zic3***(C)***, and C80913***(D)**. The Enh and PrL probabilities of 1 kb bins are shown in red and blue bars, respectively. Only bins with probabilities greater than 0.8 are displayed for higher stringency, and the y-axis scale is from 0.5 to 1. Transcription factors peaks identified using SISSRs algorithms are illustrated in rectangle boxes to demonstrate overlaps of the enhancers with TFs. *mTet1*: mouse Tet1 gene; *hTET1*: human Tet1 gene aligned to mouse mm9 genome.

We further investigated other high probability enhancer clusters and identified an enhancer dense region upstream of the *miR-290* microRNA cluster. The *miR-290* cluster, though not sufficient to maintain ES cell pluripotency alone, inhibits ES cell differentiation when over-expressed [51,52]. Four of the seven identified high probability Enh regions (prob ≥ 0.8) upstream of *miR-290* overlap with MTL (Figure 7Additional file 7: Figure S6A). Interestingly, although overlaps of putative enhancers with co-MYC regions are rare (420/19200), two Enh candidates (prob = 0.9850, 1.0000) contain MYC and MYCN binding peaks.

In addition to Enh candidates overlapping co-OSN sites, we have also identified several putative enhancers with limited OSN occupancy including those shown surrounding *Tet1/U6Zic3*, and *C80913* (Figure 7). We identified seven contiguous enhancers around the *U6* small nuclear RNA, upstream of *Tet1* which overlap TF bound regions as well as a CHD7 bound region (obtained from [37]; Figure 7, S 6B). TET1 modifies methylated cytosine (5mC) by hydroxylation, generating 5-hydroxymethylcytosine (5hmC) [53], potentially the first step in DNA demethylation [54], and is involved in regulating the lineage potential of ES cells [55]. We identified two adjacent PrL candidates upstream of the annotated mouse gene which overlap the aligned human *TET1* gene (Figure 7). The upstream enhancer (prob = 0.9138) of the *Zic3* gene showed nine-fold up-regulation of luciferase activity compared to a minimal *Oct4* promoter [56], and the higher probability enhancer downstream (prob = 0.9550), overlapping one E2F1 and one CHD7 peak, has not been investigated (Figure 7, S 6C). Furthermore, the putative enhancer 10 kb upstream of *C80913* overlaps only with a CHD7 peak (Figure 7, S 6D).

### TF motif enrichment at ES cell enhancer candidates

Although our modeling approach was trained using co-OSN regions, the prediction is not limited to sites bound by these TFs. In fact, of the 1277 regions with enhancer probability ≥0.8, 522 overlap with co-OSN regions, 394 overlap with at least 4 of the other 7 TFs, 136 of which are not co-OSN. Overall 281 regions are not associated with OCT4, SOX2 or NANOG, and 97 of our Enh candidates were not associated with any of the 12 TFs. As a number of our Enh candidates do not overlap a region bound by any of the 12 TFs for which ChIP-Seq data is available in ES cells, we next investigated which TFs may be binding these regions.

To this end we carried out a supervised motif analysis using the Clover algorithm [27] (Table 2). As validation of this approach, we detected motif enrichment for critical regulatory ES cell-expressed TFs including: KLF4, SOX2, OCT4, ESRRB and STAT3, all ranked in the top 11 enriched motifs. The PSSM for NANOG was not included in the query set (because it was not available in the PSSM databases we used) and as a result was not identified as enriched. We also detected significant enrichment in motifs for several other ES cell-expressed TFs including: SP1, SOX4, ZIC2, ZIC3, RARα, NRF2, and TEAD1. The top motif enrichment candidate, SP1, is a ubiquitously expressed transcription factor essential for early embryonic development [57,58]. ZIC3, ranks in the top 27% of absolute expression in mouse ES cells, is required for maintenance of pluripotency [56], and directly activates *Nanog*[59]. Interestingly the high probability enhancer candidate upstream of *Zic3* is bound by NANOG, suggesting NANOG also directly regulates *Zic3*. NRF2, encoded by *Nfe2l2* gene, acts as a master regulator of the antioxidant response, and its deficiency results in embryonic lethality and severe oxidative stress [60]. TEAD1 binding sites have been shown to enhance reporter gene transcription in ES cells and 2-cell embryos [61]. Although retinoic acid treatment of ES cells is associated with neuronal differentiation, RAR is expressed in ES cells and ChIP-chip revealed 462 RAR target loci in ES cells, most of which are specific to ES cells [62]. We also identified motif enrichment for TFs with limited or no expression in ES cells. These TFs play essential roles in embryonic development and early differentiation. Specifically, SOX11, ZIC1 and NR4A2 play central regulatory roles for neural development and neural protection [63-66]. This suggests that either another active protein family member may have a very similar PSSM (such as SOX2 and ZIC3) or a proportion of our enhancers are poised for regulating gene expression during development [57,67-69].

**Table 2:** Motif enrichment in mouse ES cell putative enhancers

Motif	Raw score	Max Enh prob	RPKM	PSSM
SP1	339	0.8099	33.5	[12]
KLF4^#^	154	†	126.07	[12]
SOX2^#^	140	1	942.79	[12]
POU5F1^#^	128	0.9487	1318.38	[12]
SOX4	89.6	--	37.06	[70]
SOX11	86.5	0.6035	2.86	[70]
ESRRB^#^	72.6	0.9999	162.78	[12]
KLF7	61	--	4.94	[70]
ESRRA	37	--	4.02	[70]
NR4A2	33.7	--	0.03	[12]
STAT3^#^	31.6	0.5795	29.92	[12]
ZIC2	31.3	0.4291	13.76	[70]
ZIC1	29.6	0.5901	0.06	[70]
RARA	28.3	0.5909	30.28	[70]
ASCL2	25.1	--	0.72	[70]
NR2F2	19.9	0.5537	0.07	[70]
ZIC3	14	0.9550	15.27	[70]
RORA	7	0.5016	0.13	[12]
NFE2L2 (NRF2)	6.28	0.5947	68.09	[12]
RXR::RAR_DR5	4.89	--	5.34 (*Rxra*) 17.55 (*Rxrb*) 30.28 (*Rara*)	[12]
TEAD1	3.91	0.8987	66.24	[12]

The motifs of the listed TFs are significantly enriched with *p* < 0.01 in putative enhancer regions compared to random sequences drawn from PrL set, chr19 and promoter 5 kb regions using the Clover algorithm [27] with TF binding matrices reported in PSSM column Raw scores from Clover algorithm are reported. “Max Enh prob” column lists the maximum probabilities of enhancers if at least one Enh is closest to the TSS of the corresponding gene. Probabilities greater than 0.8 are underlined. RPKM column reports the absolute gene expression of the TF in mouse ES cells. † Several putative enhancer located 50 kb downstream of *Klf4* are categorized to another non protein-coding transcript. ‘#’ sign denotes the TFs with ChIP-Seq datasets in mouse ES cells.

To verify binding of transcription factors at identified motifs we used KLF4, ESRRB, and STAT3 ChIP-Seq data from Chen et al. 2008 [5]. We compared the overlap of our high probability Enh regions to TF enriched regions (defined by the top 0.5 percentile bins for KLF4, ESRRB and STAT3). We found 519, 707 and 518 of the 1277 high probability enhancers (prob≥0.8) contained KLF4, ESRRB or STAT3 enriched regions, respectively (p values <2.2 × 10^−16^) confirming the motif enrichment results for these transcription factors.

### Identified enhancers are mainly active and cell type specific

**Figure 8:**
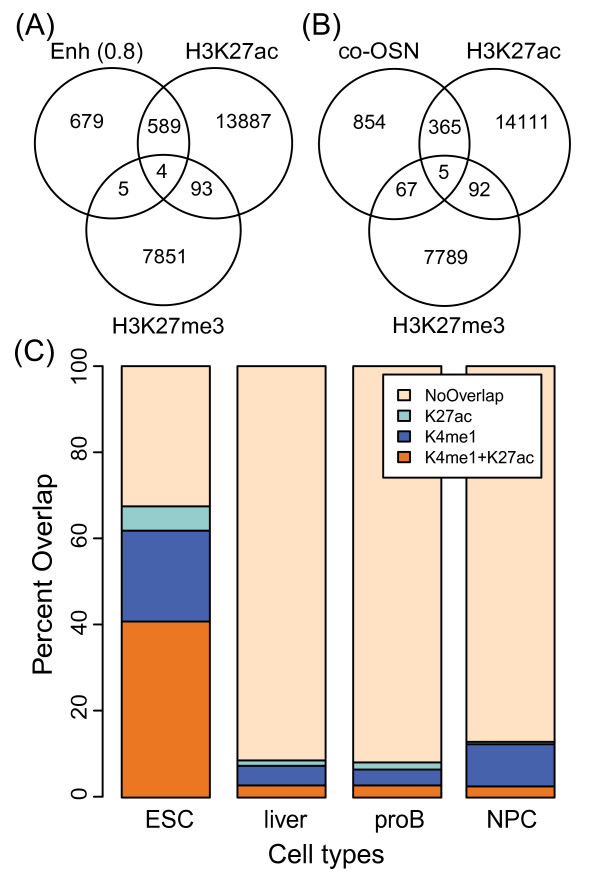
**Enhancer candidates overlap ES cell specific H3K27ac and H3K4me1 modifications.(A)** Venn diagram depicting overlap of high confidence Enh candidates (probability ≥ 0.8) with distal (TSS +/**−** 1 kb removed) H3K27ac marks (active) and H3K27me3 marks (repressive). **(B)** Venn diagram depicting overlap of OCT4, SOX2, NANOG co-bound training regions with distal H3K27ac marks (active) and H3K27me3 marks (repressive). **(C)** The stacked bar plot shows the percent overlap of high confidence Enh candidates with distal H3K27ac/H3K4me1 in various cell types. The overlaps presented allow a 500 bp gap.

Recent studies identified different classes of enhancers marked by H3K4me1 in ES cells; enhancer regions also marked by H3K27ac were associated with increased gene activity in ES cells and termed active, while regions marked by H3K27me3 were associated with early developmental genes and termed poised in ES cells [68,69]. In addition to these active and poised enhancers, another recent study proposed an intermediate category of enhancers marked by H3K4me1 but lacking H3K27ac and H3K27me3 [71]. We found significant overlap between Enh candidates (prob ≥ 0.8) from our model and the distal H3K27ac mark in ES cells (*p* < 2.2 × 10^−16^, logOdds = 3.86; Figure 8A; Additional file 8: Figure S7A). We found that 7404 of our 19200 predicted Enh regions fell into the active category while 4433 and 446 fell into the intermediate and poised categories, respectively. The top Enh candidates were significantly enriched in H3K27ac compared to the training set of co-OSN regions indicating the advantage of the model in identifying active enhancers (*p* < 2.2 × 10^−16^, logOdds = 0.77) (Figure 8B). We also increased the overlap to the H3K27ac regions compared to the training data suggesting again that we have identified additional active enhancers not contained in the training data. In addition, these candidates are significantly less associated with the repressive H3K27me3 mark compared to co-OSN regions (*p* = 3.6 × 10^−12^, logOdds = −2.1). This significant association with the H3K27ac suggests our modeling preferentially identifies active rather than poised enhancer regions. As H3K27ac overlaps with our Enh candidates and was not included in our initial model we next investigated whether or not this mark is predictive of Enh regions. Introducing H3K27ac into our model showed positive weighting of H3K27ac for PrL rather than Enh indicating that H3K27ac is a predictor of PrL rather than Enh ( Additional file 9: Figure S8).

To further investigate the cell type-specific regulatory potential of our identified ES cell Enh candidates, we compared distal H3K27ac and H3K4me1 marks from various cell types to our Enh candidates ( Additional file 8: Figure S7B). We found significantly greater Enh overlap to distal H3K27ac and H3K4me1 in ES cells compared to the differentiated cells indicating a significant proportion of our Enh candidates are specific to ES cells (*p* < 3 × 10^−5^). We observed an increase in the overlap to H3K27ac and H3K4me1 in ES cells, and a decrease in the overlap in the differentiated cell types for the high probability Enh set compared to all Enh (Figure 8C compared to Additional file 8: Figure S7B).

## Discussion

We present a systematic approach to assess discriminative features for enhancer identification in mouse ES cells. We initially established the importance of feature selection using a Naive Bayes classifier and subsequently used LASSO regularized multinomial logistic regression to systematically rank the feature weights. In our model we identified 10 key signatures for distinguishing enhancer regions from promoter-like regions and the rest of the genome. The top signatures predictive of enhancers are p300, H3K4me1, MED12, and NIPBL whereas we identified CpG islands, RNAPII-ser5, CTCF, GC percent and H3K4me3 as the top signatures for promoter identification. Our model predicts previously validated enhancers as well as novel enhancers around *Oct4*, *Sox2*, *Nanog*, *Phc1*, *Lefty1*, *Lefty2*, *miR290*, *Tet1*, and *Zic3*, all of which have important regulatory roles in regulating ES cell pluripotency. In summary, our predicted enhancers appear to regulate the expression of ES cell-expressed genes, coordinately with predicted promoters, are significantly associated with MTL compared to both PrL and unknown regions, enriched in motifs for pluripotency associated TFs and marked by tissue-specific chromatin modifications ( Additional file 10: Table S2).

The features used in our model for enhancer identification are p300, H3K4me1, MED12 and NIPBL, ranked in that order. We identified p300 as the top ranked enhancer signature; in addition p300 is a strong negative predictor for the unknown regions of the genome in our model. In ES cells p300 has been identified as a major H3K27 acetyltransferase [72]; therefore, it is not surprising that we found a high degree of overlap between our Enh candidate regions and H3K27ac. Although p300, when used as the sole Enh feature, ranked highly with respect to precision of enhancer identification the ability to recall enhancers in the training data was poor, in agreement with p300 being an incomplete representation of enhancers [24](Figure 1, rank 20). The incorporation of CBP, also linked to H3K27ac in ES cells, into the model may allow for improved enhancer identification [21]. H3K4me1, ranked second for enhancer identification, has been used in several other studies to identify enhancer regions, though we find it performs quite poorly when used as the only enhancer feature (Figure 1, rank 27). This is perhaps due to the broader regions associated with H3K4me1 compared to the more discrete peaks of p300 or MED12.

MED12 and NIPBL are the third and fourth most predictive features for enhancer identification. MED12 is part of the mediator complex and NIPBL is associated with cohesin complex loading; many components of these complexes have recently been identified to co-occupy enhancers in mouse ES cells [22]. It is somewhat surprising that MED12 and NIPBL stand out among the other mediator and cohesin components included in the model (MED1, SMC1A, SMC3) as peaks of all five are apparent at key enhancers [22]. The mediator complex is recruited by many TFs and acts as a bridge to the RNAPII preinitiation complex [22,73]. MED1 is part of the core mediator module while MED12 is part of the mediator kinase module [74,75]. The mediator core, when associated with the kinase module has been implicated as a transcriptional repressor; however, MED12 has been shown to be required for transcriptional activation by specific transcription factors, including NANOG [76]. The Drosophila homologue of NIPBL (Nipped-B) has been shown to support enhancer-promoter communication between distant enhancers [77,78]. Members of the cohesin complex, SMC1A and SMC3, in addition to being associated with enhancer regions, are also found at CTCF occupied regions while the cohesin loading factor NIPBL is less associated with CTCF [22]. CTCF is predictive of PrL in our model, which would account for SMC1A and SMC3 being less discriminatory of Enh and PrL than NIPBL.

As individual features are associated with enhancers and promoters to various degrees, the modeling approach is important to discriminate their relative contribution to enhancers and promoters. The importance of feature extraction when using chromatin signatures has been demonstrated previously using an artificial neural network for enhancer prediction in human ES cells [79]. In addition, models integrating multiple data sources, including histone modification ChIP-Seq data, have been shown to successfully improve cell type-specific TF binding site prediction [36,80]. These studies used all histone modification ChIP-Seq data available for either human or mouse ES cells to predict TF binding sites without assessing the predictive value of each feature. Narlikar et al. 2010 employed LASSO regularization to identify heart-specific enhancers by using TF binding specificities from PSSMs as features [81], our approach in contrast used non-TF chromatin-associated features from ChIP-Seq data to identify enhancers. Other unsupervised approaches have been used to systematically annotate various functional elements in the human genome using chromatin features [82,83] while our approach focused specifically on enhancer identification.

In using minimal features (8 sequencing data sets and 2 genomic features) and avoiding the use of cell-type specific TF signatures in the modeling we have retained the potential to apply this model to other cell types. The features used (p300, H3K4me1, MED12 and NIPBL for Enh; CpG islands, RNAPII-ser5, CTCF, GC percent and H3K4me3 for PrL) are genomic sequence features, ubiquitously expressed proteins, and histone modifications which we expect mark enhancers and promoters in all cell types. In fact, p300 has been used to identify enhancers in several different cells types. In contrast to methods identifying enhancers using p300, alone or in combination with H3K4me1, in ES cells we found that our enhancer candidates overlapped the active H3K27ac mark but not the repressive H3K27me3 mark [68,69,71]. This significant association with H3K27ac, and the absence of the H3K27me3 mark in our enhancer candidates indicates that the enhancers we identified are mainly of the active and intermediate, but not poised type. This conclusion is further supported by the finding that genes associated with Enh in addition to PrL showed the highest expression levels in mouse ES cells. These findings also suggest that the TFs identified in motif analysis which are associated with differentiated cell types may have been the result of similarity in PSSMs between members of the same protein family such as SOX2, SOX4 and SOX11.

Despite the reported association of H3K27ac with active enhancers, introducing H3K27ac into our model showed positive weighting of H3K27ac for PrL rather than Enh, indicating that it is a predictor of promoters rather than enhancers. This is in agreement with higher enrichment overall of H3K27ac regions in PrL compared to Enh; whereas, the previously reported H3K27ac regions [68,69] contain only the H3K27ac regions distal to genes and are therefore more enriched in the Enh category. This dichotomy of H3K27ac marks is similar to RNAPII-ser5, H3K4me3 and H3K4me2 which have been observed at distal enhancer regions (Figure 6, S 6) [20,84,85]; however, these features are more enriched around gene TSSs compared to enhancers. As our model detects the overall trend, features enriched in both PrL and Enh either become a discriminating feature of the signature in which they are most enriched or their weights are reduced to zero with LASSO regularization. It is possible that some of this overlap in marks is due to chromatin looping events which bring distal enhancers into close proximity with gene promoters [22,86]. This close juxtaposition could then allow for cross linking events between proteins at the promoter and enhancer regions capturing the marks in both locations. In addition chromatin modifying proteins recruited to either the promoter or a distal enhancer could act at both locations in the genome when they are juxtaposed in a chromatin loop.

We have shown that Enh candidates are significantly further from gene TSSs than PrL candidates, and the absolute expression distribution of genes associated with Enh and PrL candidates is significantly higher than that of genes associated with PrL alone, indicating additional activation of gene expression by Enh candidates. These results are consistent with the enhancer/promoter DNA looping model which promotes cell-type specific gene activation [3,4,6,10,22,87,88]. Furthermore, we have also found that putative enhancer regions identified in our work are significantly enriched with MTL and that higher probability Enh are associated with an increased number of bound TFs. This finding reveals the utility of our model in identifying high confidence regions bound by multiple TFs which are more likely associated with gene regulation [5,24]. Moreover, as only 41% of the top candidates are co-bound by OCT4, SOX2 and NANOG, enhancers identified from our approach are not limited to the training set of co-OSN regions. To further identify novel enhancer-bound TFs that may play major roles in mouse ES cells, we performed motif enrichment analysis on enhancer candidates. Although motif enrichment analysis is limited by the PSSM available, we have successfully identified motif enrichment of several known ES cell-regulating TFs, including: KLF4, SOX2, OCT4, ESRRB, STAT3 and ZIC3. In addition, we identified SP1, previously reported to regulate *Oct4* and *Nanog* gene expression through binding to their proximal promoters [89,90]. Binding motifs for NRF2 and TEAD1 were also identified as enriched in the Enh regions. Both of these TFs are associated with regulatory roles during early development and are expressed in ES cells [60,61]. Interestingly, we have also identified enhancer regions closest to genes of almost all of these regulatory TFs. In agreement with this we found that genes associated with Enh candidates are more exclusively enriched in GO terms related to DNA binding and transcriptional regulation, while the PrL genes are associated with DNA binding as well as more basal cellular functions. Together these findings suggest that enhancers tend to locate around genes involved in transcriptional regulation in ES cells, and work coordinately with PrL candidates. The combination of regulation by a distal enhancer and proximal basal promoter perhaps allows gene expression to be fine tuned in a cell-type specific manner.

## Conclusions

We identified a widely applicable set of features to identify regulatory enhancers and promoter regions in a given cell type. Use of these features yields enhancer regions associated with increased gene expression of neighbouring genes and ES cell-specific histone modifications consistent with active enhancers.

## Methods

The bioinformatics analyses were done in R 2.12.0 (http://www.r-project.org/) and Bioconductor [91] unless otherwise stated.

### Feature and TF datasets

Thirty public domain ChIP-Seq raw data sets in mouse ES cells were obtained from Gene Expression Omnibus (GEO) [92]: 12 TFs [5], 8 histone modifications [29], 3 polymerase occupancy [30] and 7 chromatin associated proteins [5,22,28] (Table 1). Although not listed in the table, five ChIP-Seq controls corresponding to the above features were also downloaded. Processed RNA-Seq data from Guttman et al. 2010 was obtained for expression analysis [32]. Other genomic features such as Phastcons most conserved regions [31,33], CpG islands [34], GC contents, SNP and repeats were downloaded from UCSC genome annotation database in mouse mm9 build [35]. We used Ensembl transcripts for gene annotation and UCSC Genes for figure illustration.

### Data pre-processing

Reads from ChIP-Seq data were aligned to mouse mm9 assembly using Bowtie alignment [93] by suppressing alignments to only 1 best reportable alignment with a maximum number of 2 mismatches within 28 nucleotides in the high quality sequencing end. The mouse genome was segmented into 1 kb bins, and target tag count within each bin was normalized by dividing by the control tag count plus 3 (the rounded average median tag count within a bin for all ChIP-Seq data), to reduce the effect of low input count in generating extreme ratios. Format conversion was done using the Vancouver Short Read Analysis Package (http://vancouvershortr.sourceforge.net/). The same procedure was done for all ChIP-Seq data sets to obtain a vector of values for each protein or TF. While ChIP-Seq data files for each TF in the same experimental setting were combined, exact duplicate tags were removed to avoid PCR amplification bias generated in the sequencing library preparation. As the control ChIP-Seq data sets were not uniformly distributed throughout the genome, 180 bins with read counts in top 0.5 percentile for all 5 controls were first excluded from the analysis. The genomic features were subsequently quantified in each bin using counts without normalization, eg. number of CpG islands within each bin. In enhancer candidate plots, significant TF binding peaks (*p* < 0.001) predicted with SISSRs algorithm (v1.4) [94] are labeled in order to show binding peaks defined from another source.

### Training data sets

Binding regions of each TF used as training data were defined to be regions with number of tags in the top 0.5 percentile. As ChIP-Seq co-bound regions of the pluripotency transcription factors OCT4, SOX2 and NANOG were confirmed to have enhancer activity in 25/25 cases and ChIP-Seq co-bound regions of MYC or MYCN were shown to have very weak or no ES-cell-specific enhancer activity in luciferase assays [5], these co-bound regions were used as Enh and PrL training sets respectively using TF independent features. More specifically, 1291 co-bound regions of OCT4/SOX2/NANOG (co-OSN) without either of MYC/MYCN binding were taken as the Enh training set; whereas 4465 co-bound regions of MYC and MYCN (co-MYC) without either of OSN were taken as the PrL training set. Due to the promoter-like nature of the co-MYC cluster (Figure 1a), 5000 random regions were drawn from non co-OSN and non co-MYC regions to be the third category called ‘unknown’.

### Feature combination assessment using Naive Bayes

Naive Bayes classifiers with various feature combinations were used to classify 1 kb genome bins into the three categories. Due to limited amount of training data, 10-fold cross validation was performed to the training data set by randomly leaving 10% of the data as a validation set. Classifier assessment was carried out using the mean of area under curve (AUC), precision, modified precision, and recall values computed from the validation set. AUC is the area under ROC curve. Precision, normally given by true positive over sum of true positive and false positive, was modified to report the percentage of co-OSN regions out of putative enhancers that were originally Enh or PrL. This was done to avoid penalizing potential Enh candidates in the unknown validation set. Recall, given by true positive over sum of true positive and false negative, measures the percentage of all co-OSN regions predicted to be enhancers. These indices provide different aspects on model assessment. Ranking of the classifiers with different feature combinations were sorted by the average ranking of all indices.

### Feature extraction and weighting with LASSO regularized multinomial logistic regression

All the features were first standardized by subtracting the mean and dividing by the standard deviation in order to prevent biased shrinkage of feature weights. Multinomial logistic regression was applied to model sequence features, chromatin features, and associated proteins to predict the genome-wide location of enhancers. Cross entropy was used as the error function for multi-group classification. To assess predictability of features, LASSO regularization was used to introduce extra penalization with a power raised on the weight vector [25,26]. Using LASSO regularization, feature weights of less significance shrink to 0 as lambda increases and the lambda, exp(−4), is subsequently obtained through comparison of average multinomial deviances from 10-fold cross validation.

### Correlations of Enh and PrL sets with absolute gene expression in mouse ES cells

RPKM, the number of reads per kilobase of exon region per million mapped reads derived from RNA-Seq data has been shown to be approximately proportional to the absolute abundance of mRNAs in cell [95]. We obtained formerly computed RPKM values by Ouyang et al. [96] from a mouse ES cell RNA-Seq data set [97]. The Enh and PrL candidates were assigned to Ensembl genes of the closest TSSs without taking the degree of transcription into account. The distributions of absolute expression (RPKM) were shown in box plots, and one-sided Kolmogorov-Smirnov tests were performed to assess the differences in empirical cumulative distributions of gene sets.

### GO functional enrichment analysis and MTL association

Candidates were first assigned to genes through identifying the closest TSSs. 469 Enh candidates (Enh prob ≥ 0.9) and 2239 PrL candidates (PrL prob ≥ 0.99) were used to carry out the analysis. The discrepancy in probability cut-offs between sets was due to the large number of high confidence PrL candidates. DAVID functional annotation website was used to assess functional enrichment on both Enh and PrL candidate sets compared to all genes in the mouse genome [42,43]. As genes can have more than one nearby candidate within each set, only the unique genes within each set were subjected to GO analysis. The molecular functions and biological processes significantly enriched in each set were subsequently reported (FDR < 0.1). For better visualization of the enrichment functions, Enrichment map plug-in of Cytoscape was used to cluster functions sharing the same genes [44,98].

To determine if the putative enhancer candidates are enriched in MTL, we separate Enh, PrL and unknown candidates, and all genome bins into 3 groups: 0 TF peak enrichment, enrichment of 1 to 3 TFs, and enrichment of over 4 TFs (MTL) within the bin. The MTL enrichment of Enh is assessed using Chi-squared statistics of the counts of the last two bins in comparison to that of PrL, unknown and genomic bin sets.

### Supervised motif analysis

Clover algorithm [27] was used to screen a set of given DNA sequences against transcription factor weight matrices, and assess whether any motifs are over- or under-represented in the given sequences by comparing to random sequences drawn from chr19 (42.7% C + G) and 5 kb upstream of TSSs (45.7% C + G). For a more stringent motif screening, we also tested the significance of the Enh regions compared to 2222 PrL regions (PrL prob > 0.99; 61% C + G). Motif and sequence shuffling were also used to account for G + C content biases. 467 putative enhancer regions with Enh probability greater than 0.9 (45% C + G) were used in the motif analysis to narrow down the search. We obtained the human and mouse PSSMs from the JASPAR mammalian database [12] and mouse protein-binding microarray data [70].

### Binding region comparison with other datasets

Significantly enriched regions of H3K4me1, H3K27ac and p300 in ES, adult liver, progenitor B and neural progenitor cells were obtained from [69], and genomic coordinates were updated to mm9 using the UCSC genome browser liftOver tool [35]. 10479 and 2916 CHD7 binding peaks from medium and high thresholds were obtained from [37], and the genome coordinates were also updated to mm9 built. Venn diagrams are drawn on the basis of putative enhancers allowing 500 bp gaps between regions.

## Abbreviations

ES cell: Embryonic stem cell; co-OSN: OCT4 SOX2, and NANOG ChIP-Seq co-bound; co-MYC: MYC and MYCN ChIP-Seq co-bound; TF: Transcription factor; PSSM: Position-Specific Scoring Matrix; MTL: Multiple transcription factor-bound loci; TSS: Transcription start site; RNAPII: RNA polymerase II; Enh: Enhancer; PrL: Promoter-like.

## Competing interests

The authors declare that they have no competing interests.

## Authors’ contributions

JAM and CYC conceived and designed the study. QM helped supervise the computational analysis. CYC conducted all experiments. JAM and CYC interpreted the results. CYC and JAM wrote the manuscript which all authors approved.

## Supplementary Material

Additional file 1 **Table S1. Predicted enhancer and promoter-like candidates.** The Enh and PrL candidates in mouse ES cells are listed. Columns represent the chromosome location (Chr, start and end), Enh / PrL probability (Enh.prob / PrL.prob), gene symbol of the closest Ensembl transcript (geneName), the location relative to genes (location), the distance of the enhancer to the closest transcript (distance2Transcript), the previously validated enhancers that overlap with Enh from Chen *et al*. 2008 (Validated), and whether the candidate overlaps an exon (OverlapExon). Click here for file

Additional file 2 **Figure S1. Heatmap of features used in LASSO regression for top 50 enhancer and promoter-like candidates.** The dark red and blue side bar on the left denotes putative Enh and PrL 1kb genome bins, whereas the dark red and blue side bar on top denotes indicative Enh and PrL feature sets. Feature values are scaled to exhibit the contrast between the Enh and PrL. Click here for file

Additional file 3 **Figure S2. Genomic distribution of categories and relative position to TSS (The entire set).** (A) Pie charts representing the genomic distributions of the co-OSN and co-MYC training sets as well as all Enh and PrL candidates. Intergenic regions are defined to be regions ≥ 10kb away from the closest TSS or transcription end site; whereas upstream regions are regions within 10kb upstream of TSSs. (B) Violin plots demonstrating the distances to TSSs of the closest transcript for each set. Click here for file

Additional file 4 **Figure S3. Gene Ontology analysis of the Enh and PrL candidate sets.** Enriched functions of Enh (A) and (B) PrL identified from DAVID (FDR<0.1) and plotted using Cytoscape Enrichment map plug-in. Functions are further circled and grouped into general categories labeled aside. Line thickness between nodes is proportional to number of genes shared between nodes. Colors are used for the purpose of visualization contrast between functional groups. Click here for file

Additional file 5 **Figure S4. Four known mouse ES cells enhancers that interact with nearby promoters through looping mechanisms.** Plot showing previously validated enhancers around *Pou5f1* (*Oct4*), *Nanog*, *Phc1*, and *Lefty1*. The Enh and PrL probabilities of 1kb bins are shown in red and blue bars, respectively. Only probabilities greater than 0.8 are shown for higher stringency (n=1277 for Enh; n=21581 for PrL), and the y-axis scale is from 0.5 to 1. Transcription factors peaks identified using the SISSRs algorithm are illustrated in rectangle boxes to demonstrate overlaps of the enhancers with TFs. Click here for file

Additional file 6 **Figure S5. LincRNA downstream of Sox2.** Plot showing novel putative enhancers downstream of the *Sox2* gene. The Enh and PrL probabilities of 1kb bins are shown in red and blue bars, respectively. Only bins with probabilities greater than 0.8 are displayed for higher stringency, and the y-axis scale is from 0.5 to 1. A lincRNA approximately 100kb downstream of Sox2 near a distal enhancer cluster is shown. Transcription factors peaks identified using the SISSRs algorithm are illustrated in rectangle boxes to demonstrate overlaps of the enhancers with TFs. The coverage plot for RNA-Seq data in ES cells is shown at the bottom. Click here for file

Additional file 7 **Figure S6. Detailed plots for novel putative enhancer regions.** Detailed coverage plots of novel enhancer regions identified including (A) multiple putative enhancers upstream of *miR-290 cluster*, (B) multiple contiguous enhancer regions upstream of *Tet1* and around a non-coding small nuclear RNA, *U6*, (C) two putative enhancers around *Zic3,* and d) the putative enhancer region located 10kb upstream of *C80913*. The Enh and PrL probabilities of 1kb bins are shown in red and blue bars, respectively. Only bins with probabilities greater than 0.8 are displayed for higher stringency, and the y-axis scale is from 0.5 to 1. Transcription factors peaks identified using the SISSRs algorithm are illustrated in rectangle boxes to demonstrate overlaps of the enhancers with TFs. Click here for file

Additional file 8 **Figure S7. Active enhancers and cell specificity of all enhancer candidates.** (A) Venn diagrams of all enhancer candidates with distal (TSS +/- 1kb removed) H3K27ac marks (active) and H3K27me3 marks (repressive). (B) The stacked bar plot shows the percent overlaps of all enhancers with distal H3K27ac / H3K4me1 in various cell types. All overlaps presented here allow a 500 bp gap. Click here for file

Additional file 9 **Figure S8. Feature coefficients determined from Lasso regularization (H3K27ac included).** The plot shows feature weights in each class with respect to logged lambda, the penalization parameter, in LASSO regularized multinomial logistic regression. Weights of features less discriminative of the three categories shrink to 0 as the lambda is increased. H3K27ac, a positive predictor of PrL group, is highlighted in a blue box. Click here for file

Additional file 10 **Table S2. Features found at predicted enhancer and promoter-like candidates.** The numbers of predicted Enh and PrL regions that overlapped with the indicated features are shown. Both the full set of predictions and the probability >0.8 set are shown as well as the overlaps with the unknown regions. P300, H3K4me1 and H3K27ac data are from Creyghton et al. 2010, CHD7 data is from Schnetz et al. 2010. * Data not shown as peaks overlapping TSS were removed from Creyghton et al. 2010 data and PrL frequently overlap TSS. Transcription factor peaks includes: KLF4, STAT3, SMAD1, E2F1, TFCP2L1, ZFX, and ESRRB ChIP-Seq data from mouse ES cells. LincRNA (large intergenic non-coding RNA). Click here for file
