# Supplementary material for: Enhancer identification in mouse embryonic stem cells using integrative modeling of chromatin and genomic features
Source: BMC Genomics. 2012 Apr 26;13:152. doi: 10.1186/1471-2164-13-152 (PMC3406964; doi:10.1186/1471-2164-13-152)
Supplement: Additional file 2 — Figure S1. Heatmap of features used in LASSO regression for top 50 enhancer and promoter-like candidates. The dark red and blue side bar on the left denotes putative Enh and PrL 1kb genome bins, whereas the dark red and blue side bar on top denotes indicative Enh and PrL feature sets. Feature values are scaled to exhibit the contrast between the Enh and PrL. [file 1471-2164-13-152-S2.pdf]

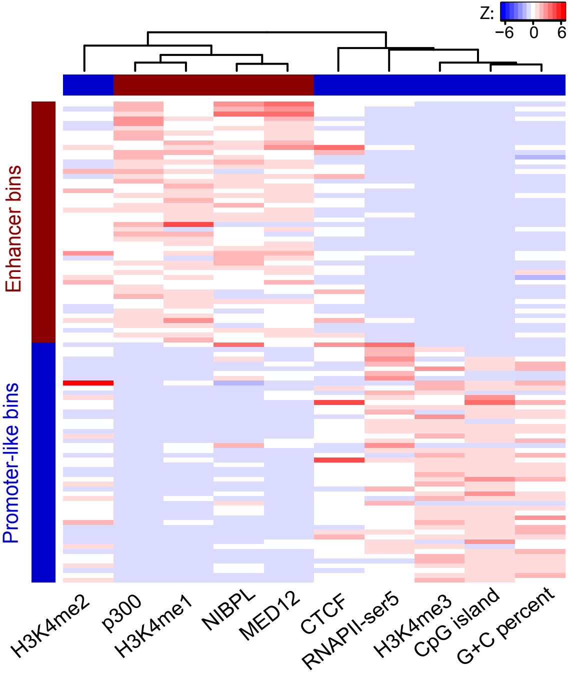
Z:
−6 0 6
Enhancer bins
Promoter-like bins
H3K4me2
p300
H3K4me1
NIBPL
MED12
CTCF
RNAPII-ser5
H3K4me3
CpG island
G+C percent
