# Supplementary figures and images for: Enhancer identification in mouse embryonic stem cells using integrative modeling of chromatin and genomic features
Source: BMC Genomics. 2012 Apr 26;13:152. doi: 10.1186/1471-2164-13-152 (PMC3406964; doi:10.1186/1471-2164-13-152)

(A)

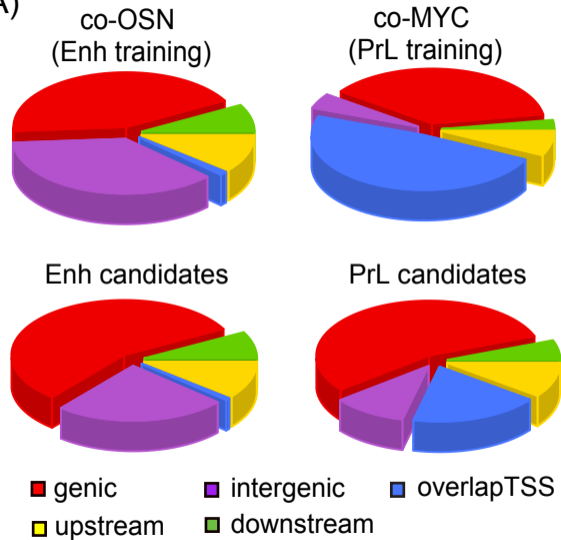

(B)

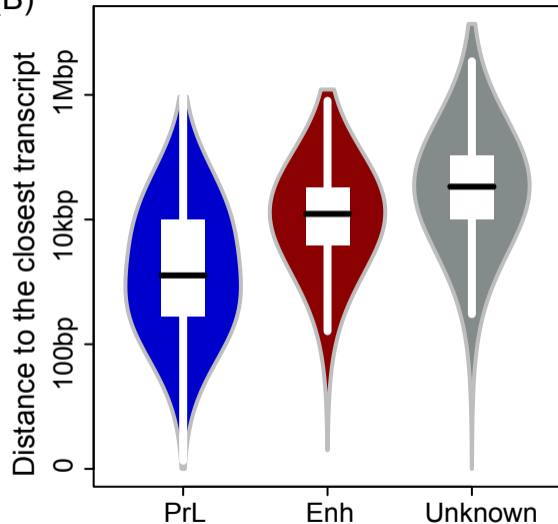

Supplement: Additional file 3 — Figure S2. Genomic distribution of categories and relative position to TSS (The entire set). (A) Pie charts representing the genomic distributions of the co-OSN and co-MYC training sets as well as all Enh and PrL candidates. Intergenic regions are defined to be regions ≥ 10kb away from the closest TSS or transcription end site; whereas upstream regions are regions within 10kb upstream of TSSs. (B) Violin plots demonstrating the distances to TSSs of the closest transcript for each set. [file 1471-2164-13-152-S3.pdf]

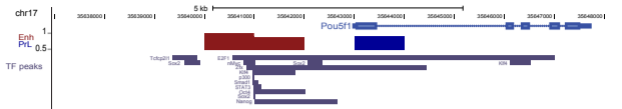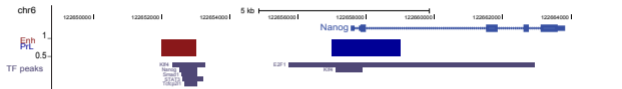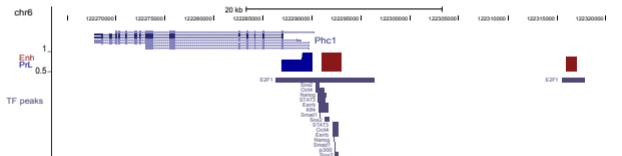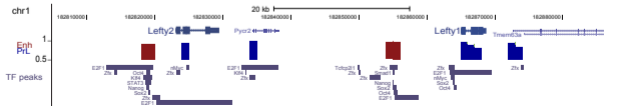

Supplement: Additional file 5 — Figure S4. Four known mouse ES cells enhancers that interact with nearby promoters through looping mechanisms. Plot showing previously validated enhancers around Pou5f1 (Oct4), Nanog, Phc1, and Lefty1. The Enh and PrL probabilities of 1kb bins are shown in red and blue bars, respectively. Only probabilities greater than 0.8 are shown for higher stringency (n=1277 for Enh; n=21581 for PrL), and the y-axis scale is from 0.5 to 1. Transcription factors peaks identified using the SISSRs algorithm are illustrated in rectangle boxes to demonstrate overlaps of the enhancers with TFs. [file 1471-2164-13-152-S5.pdf]

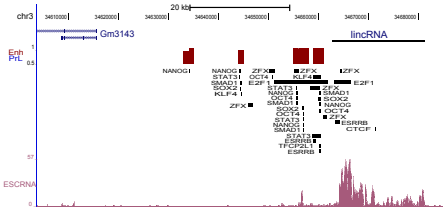

Supplement: Additional file 6 — Figure S5. LincRNA downstream of Sox2. Plot showing novel putative enhancers downstream of the Sox2 gene. The Enh and PrL probabilities of 1kb bins are shown in red and blue bars, respectively. Only bins with probabilities greater than 0.8 are displayed for higher stringency, and the y-axis scale is from 0.5 to 1. A lincRNA approximately 100kb downstream of Sox2 near a distal enhancer cluster is shown. Transcription factors peaks identified using the SISSRs algorithm are illustrated in rectangle boxes to demonstrate overlaps of the enhancers with TFs. The coverage plot for RNA-Seq data in ES cells is shown at the bottom. [file 1471-2164-13-152-S6.pdf]

(A)

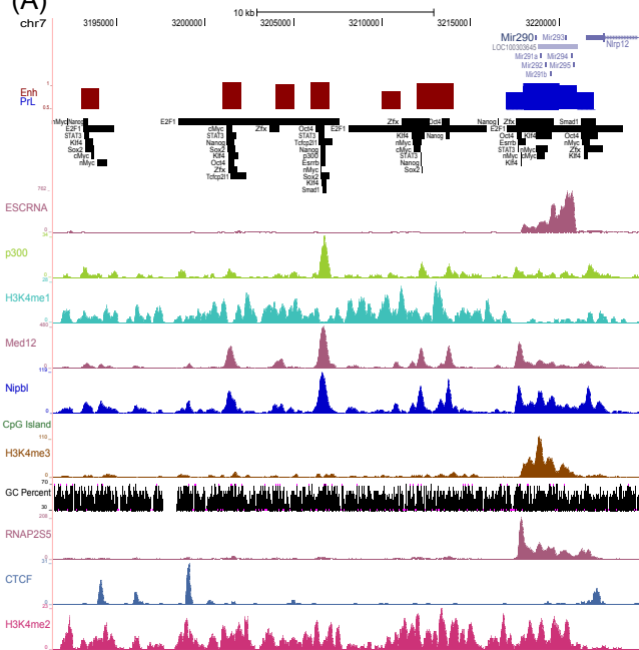

(B)

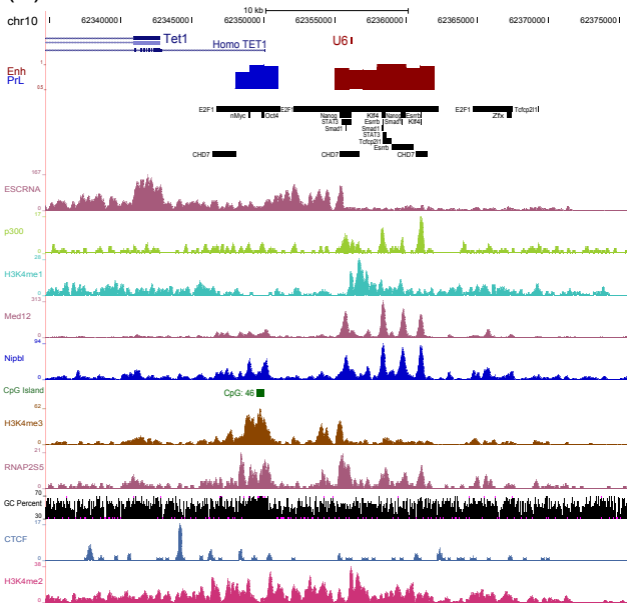

(C)

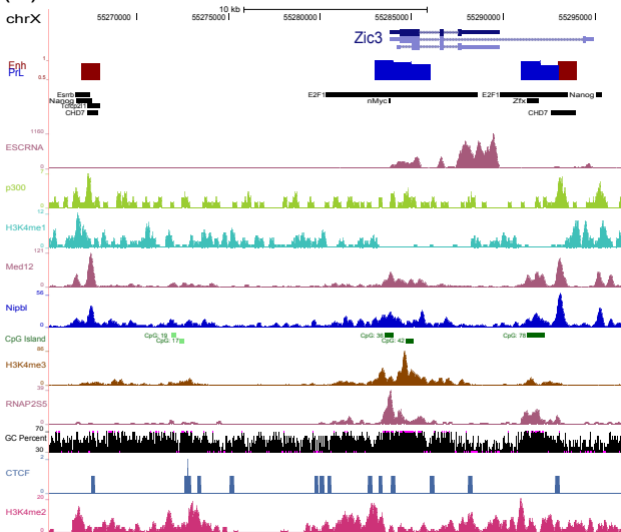

**(D)**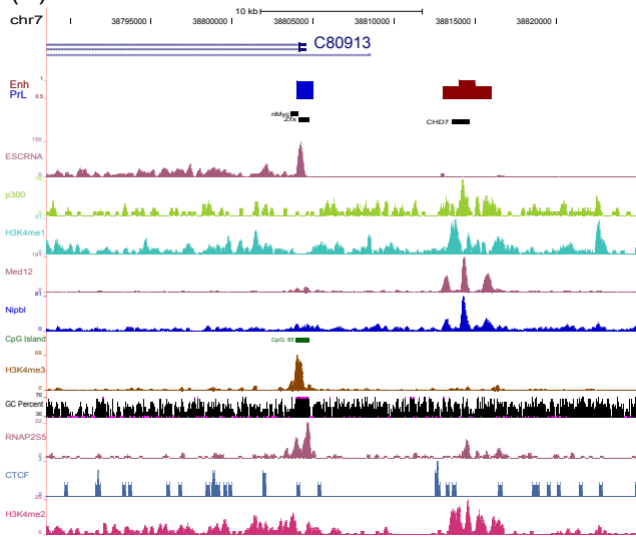

Supplement: Additional file 7 — Figure S6. Detailed plots for novel putative enhancer regions. Detailed coverage plots of novel enhancer regions identified including (A) multiple putative enhancers upstream of miR-290 cluster, (B) multiple contiguous enhancer regions upstream of Tet1 and around a non-coding small nuclear RNA, U6, (C) two putative enhancers around Zic3, and d) the putative enhancer region located 10kb upstream of C80913. The Enh and PrL probabilities of 1kb bins are shown in red and blue bars, respectively. Only bins with probabilities greater than 0.8 are displayed for higher stringency, and the y-axis scale is from 0.5 to 1. Transcription factors peaks identified using the SISSRs algorithm are illustrated in rectangle boxes to demonstrate overlaps of the enhancers with TFs. [file 1471-2164-13-152-S7.pdf]

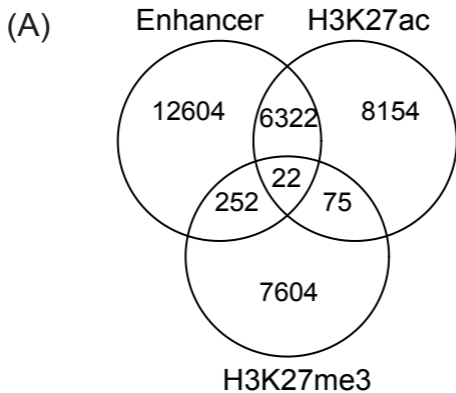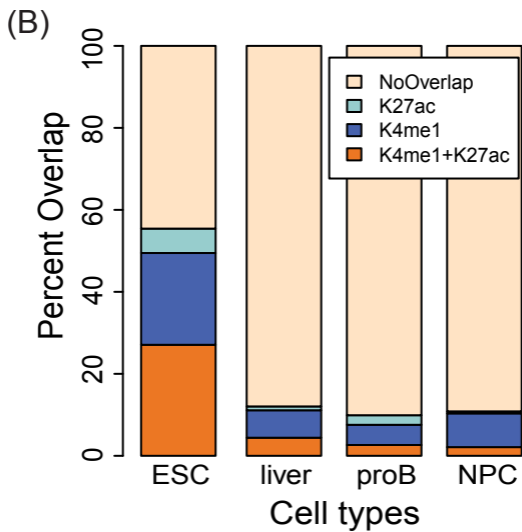

Supplement: Additional file 8 — Figure S7. Active enhancers and cell specificity of all enhancer candidates. (A) Venn diagrams of all enhancer candidates with distal (TSS +/- 1kb removed) H3K27ac marks (active) and H3K27me3 marks (repressive). (B) The stacked bar plot shows the percent overlaps of all enhancers with distal H3K27ac / H3K4me1 in various cell types. All overlaps presented here allow a 500 bp gap. [file 1471-2164-13-152-S8.pdf]

Feature weights

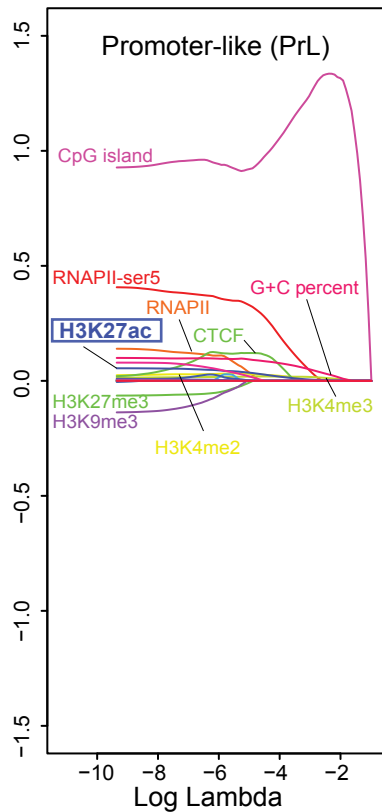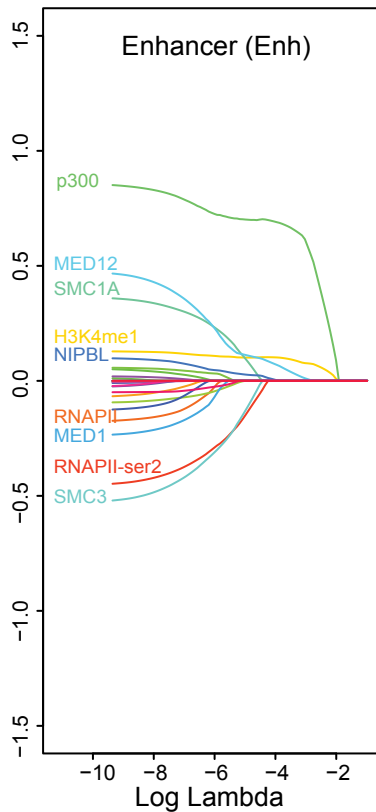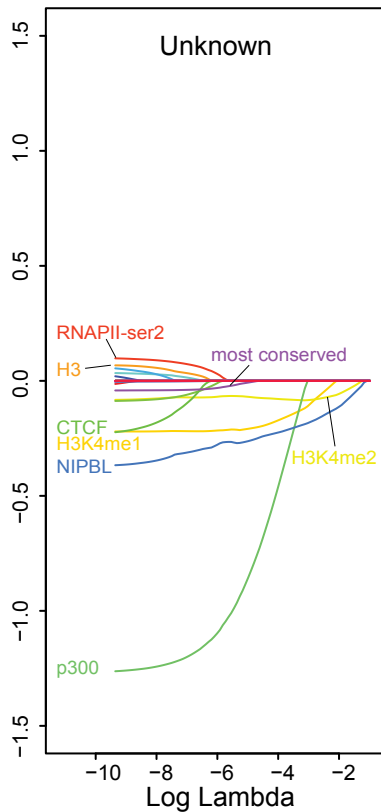

Supplement: Additional file 9 — Figure S8. Feature coefficients determined from Lasso regularization (H3K27ac included). The plot shows feature weights in each class with respect to logged lambda, the penalization parameter, in LASSO regularized multinomial logistic regression. Weights of features less discriminative of the three categories shrink to 0 as the lambda is increased. H3K27ac, a positive predictor of PrL group, is highlighted in a blue box. [file 1471-2164-13-152-S9.pdf]
