# Supplementary material for: Enhancer identification in mouse embryonic stem cells using integrative modeling of chromatin and genomic features
Source: BMC Genomics. 2012 Apr 26;13:152. doi: 10.1186/1471-2164-13-152 (PMC3406964; doi:10.1186/1471-2164-13-152)
Supplement: Additional file 4 — Figure S3. Gene Ontology analysis of the Enh and PrL candidate sets. Enriched functions of Enh (A) and (B) PrL identified from DAVID (FDR<0.1) and plotted using Cytoscape Enrichment map plug-in. Functions are further circled and grouped into general categories labeled aside. Line thickness between nodes is proportional to number of genes shared between nodes. Colors are used for the purpose of visualization contrast between functional groups. [file 1471-2164-13-152-S4.pdf]

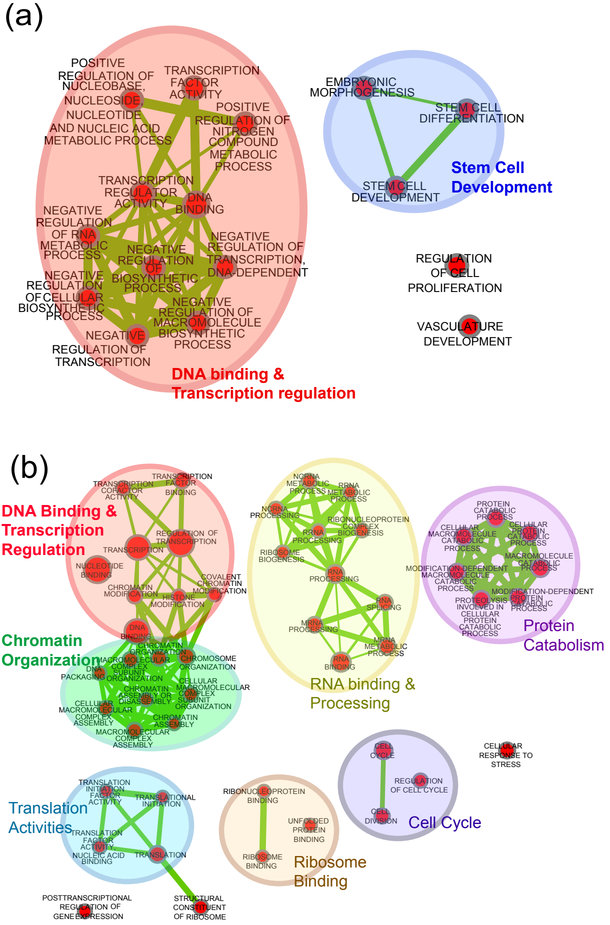
(a)
POSITIVE REGULATION OF NUCLEOBASE, NUCLEOSIDE, NUCLEOTIDE AND NUCLEIC ACID METABOLIC PROCESS
TRANSCRIPTION FACTOR ACTIVITY
POSITIVE REGULATION OF NITROGEN COMPOUND METABOLIC PROCESS
TRANSCRIPTION REGULATOR ACTIVITY
DNA BINDING
NEGATIVE REGULATION OF RNA METABOLIC PROCESS
NEGATIVE REGULATION OF BIOSYNTHETIC PROCESS
NEGATIVE REGULATION OF TRANSCRIPTION, DNA-DEPENDENT
NEGATIVE REGULATION OF CELLULAR BIOSYNTHETIC PROCESS
NEGATIVE REGULATION OF MACROMOLECULE BIOSYNTHETIC PROCESS
NEGATIVE REGULATION OF TRANSCRIPTION
DNA binding & Transcription regulation
EMBRYONIC MORPHOGENESIS
STEM CELL DIFFERENTIATION
STEM CELL DEVELOPMENT
Stem Cell Development
REGULATION OF CELL PROLIFERATION
VASCULATURE DEVELOPMENT
(b)
TRANSCRIPTION COFACTOR ACTIVITY
TRANSCRIPTION FACTOR BINDING
DNA Binding & Transcription Regulation
TRANSCRIPTION
REGULATION OF TRANSCRIPTION
NUCLEOTIDE BINDING
COVALENT CHROMATIN MODIFICATION
CHROMATIN MODIFICATION
HISTONE MODIFICATION
DNA BINDING
Chromatin Organization
CHROMATIN ORGANIZATION
MACROMOLECULAR COMPLEX SUBUNIT ORGANIZATION
CHROMOSOME ORGANIZATION
DNA PACKAGING
CELLULAR MACROMOLECULAR COMPLEX SUBUNIT ORGANIZATION
CHROMATIN ASSEMBLY OR DISASSEMBLY
CELLULAR MACROMOLECULAR COMPLEX ASSEMBLY
CHROMATIN ASSEMBLY
MACROMOLECULAR COMPLEX ASSEMBLY
NCRNA METABOLIC PROCESS
RRNA METABOLIC PROCESS
NCRNA PROCESSING
RIBONUCLEOPROTEIN COMPLEX BIOGENESIS
RRNA PROCESSING
RIBOSOME BIOGENESIS
RNA PROCESSING
RNA SPLICING
MRNA PROCESSING
MRNA METABOLIC PROCESS
RNA BINDING
RNA binding & Processing
PROTEIN CATABOLIC PROCESS
CELLULAR MACROMOLECULE CATABOLIC PROCESS
CELLULAR PROTEIN CATABOLIC PROCESS
MACROMOLECULE CATABOLIC PROCESS
MODIFICATION-DEPENDENT MACROMOLECULE CATABOLIC PROCESS
MODIFICATION-DEPENDENT PROTEIN CATABOLIC PROCESS
PROTEOLYSIS INVOLVED IN CELLULAR PROTEIN CATABOLIC PROCESS
Protein Catabolism
TRANSLATION INITIATION FACTOR ACTIVITY
TRANSLATIONAL INITIATION
Translation Activities
TRANSLATION FACTOR ACTIVITY, NUCLEIC ACID BINDING
TRANSLATION
POSTTRANSCRIPTIONAL REGULATION OF GENE EXPRESSION
STRUCTURAL CONSTITUENT OF RIBOSOME
RIBONUCLEOPROTEIN BINDING
UNFOLDED PROTEIN BINDING
RIBOSOME BINDING
Ribosome Binding
CELL CYCLE
REGULATION OF CELL CYCLE
CELL DIVISION
Cell Cycle
CELLULAR RESPONSE TO STRESS
